# Supplementary material for: A Size Filter Regulates Apical Protein Sorting
Source: Res Sq. 2023 Aug 4:rs.3.rs-3210598. Preprint. [Version 1] doi: 10.21203/rs.3.rs-3210598/v1 (PMC10418535; doi:10.21203/rs.3.rs-3210598/v1)
Supplement: Supplement 1 [file NIHPPrs3210598v1-supplement-1.pdf]

Extended Data 1

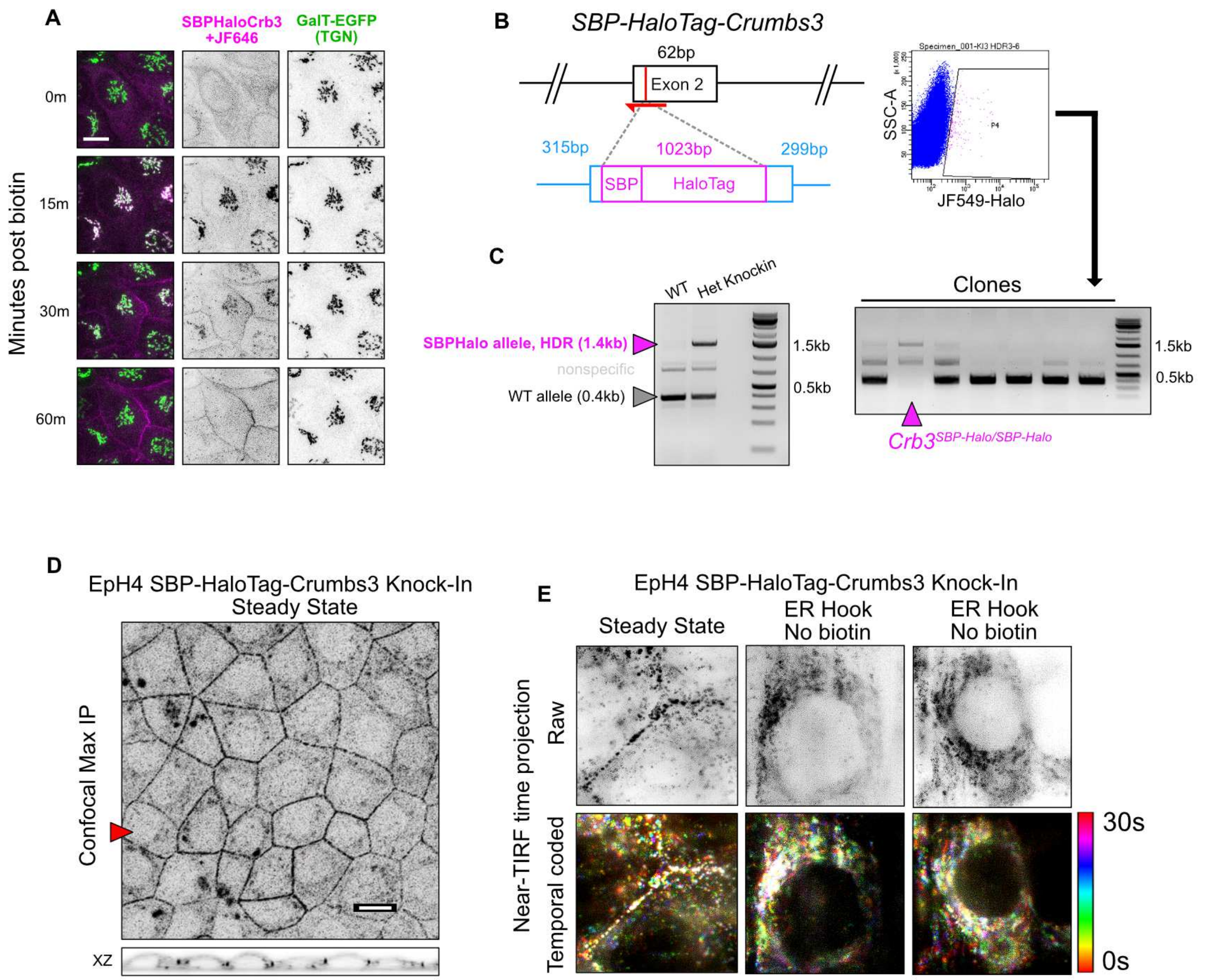

Extended Data Fig. S1.
(A) Confocal imaging of exogenous SBP-HaloTag-Crb3 RUSH in cells also expressing trans-Golgi reporter GalT-EGFP. Confocal maximum intensity projections. Scalebar 10µm.
(B) Left: Schematic of CRISPR/Cas9 gene editing strategy to insert the SBP-HaloTag sequence into *Crb3* exon 2, which encodes amino acids immediately following the signal peptide of Crb3. In red: guide RNA targeting and approximate cut site. In magenta: SBP-HaloTag insert. In blue: homology arms flanking the insert to facilitate homology directed repair (HDR). Right: FACS gating to isolate JF549-Halo+ clones.
(C) Genotyping for *SBP-Halo* HDR alleles in putative knock-in clones. PCR product lengths: wild-type allele: 385bp; HDR allele: 1408bp; nonhomologous end-joining allele: ~1700 & 2100bp (none shown).
(D) Confocal microscopy of EpH4 $Crb3^{SBP\text{-}Halo/SBP\text{-}Halo}$ cells at steady state labeled with JF549-Halo. Maximum intensity projection or XZ orthogonal view. Scalebar 10µm.
(E) Near-TIRF of EpH4 $Crb3^{SBP\text{-}Halo/SBP\text{-}Halo}$ cells at steady state labeled with JF549-Halo and imaged live, at either steady state (junctional) or with the StrKDEL hook added (ER localized). Images are maximum intensity projections in time over a 30 second span. Top row: raw data. Bottom row: temporal color coded.

Extended Data 2

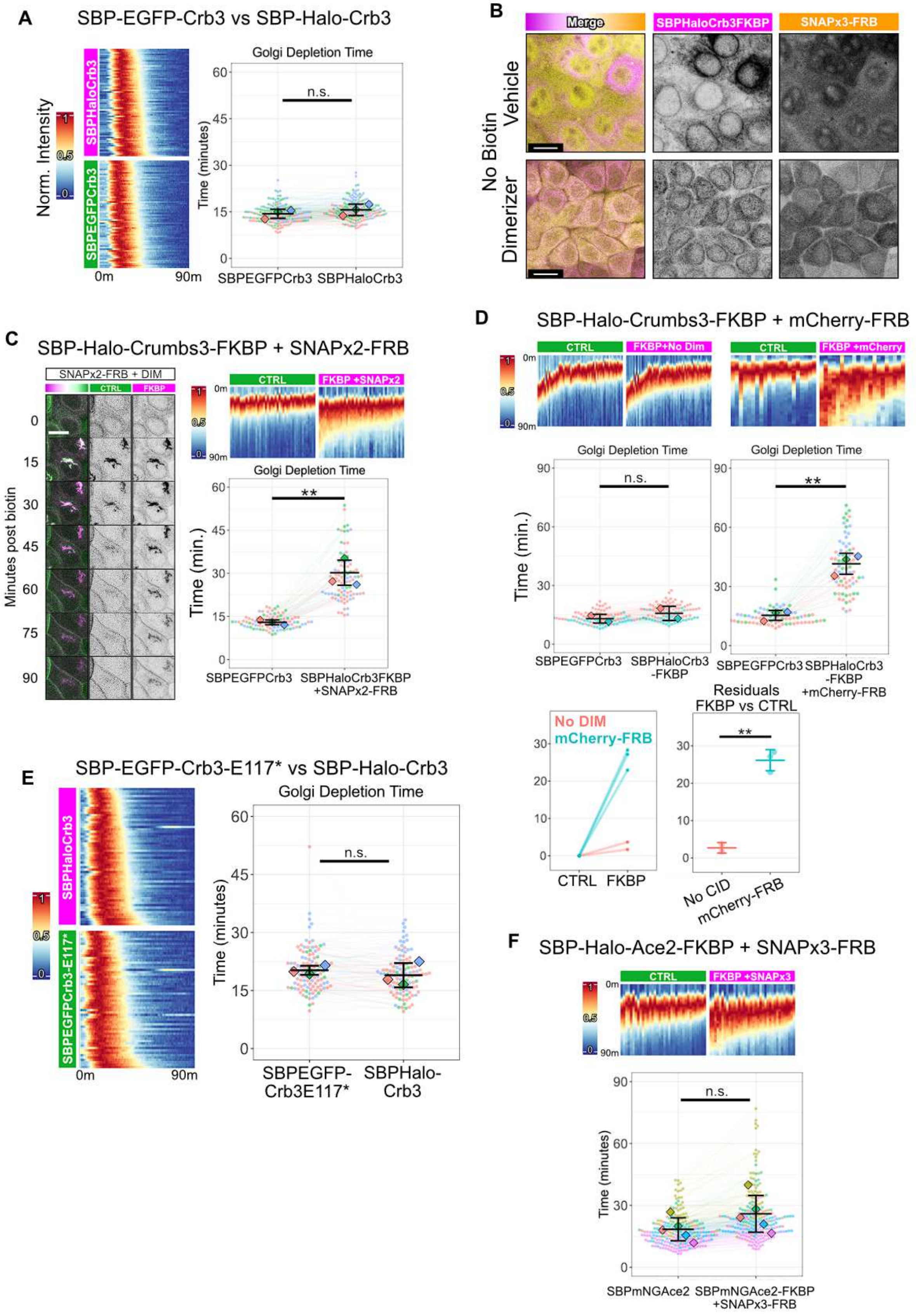

Extended Data Fig. S2.

(A) Golgi trafficking dynamics of SBP-EGFP-Crb3 versus SBP-Halo-Crb3. n = 179 total cells. N = 3 experiments. Student's t-test. n.s. p = 0.398.

(B) Visualization of SNAPtagx3-FRB in cells expressing ER-retained SBP-Halo-Crb3-FKBP, with either vehicle or with CID. Note nuclear localization in vehicle, which transitions to colocalize with the FKBP-fused construct following addition of CID.

(C) Extended data related to Fig. 3D-G, using the same methodology but with SNAPtagx2-FRB as recruitable cargo in the presence of CID. n = 90 total cells. N = 3 experiments. Student's t-test. **p = 0.00256.

(D) Extended data related to Fig. 3D-G, using the same methodology but with mCherry-FRB as recruitable cargo, and either vehicle or CID. Vehicle: n = 94 total cells. N = 2 experiments. Welch's t-test. n.s. p = 0.481. CID: n = 74 total cells. N = 3 experiments. Student's t-test. **p = 0.00155. Residuals: One-way ANOVA. **p = 0.00184.

(E) Golgi trafficking dynamics of mutant SBP-EGFP-Crb3-E117STOP, lacking the PDZ-binding motif, versus full length SBP-Halo-Crb3. n = 108 total cells. N = 3 experiments. Student's t-test. n.s. p = 0.547.

(F) Extended data related to Fig. 4C-D, using the same methodology but with SNAPtagx3-FRB as recruitable cargo in the presence of CID. n = 228 total cells. N = 5 experiments. Student's t-test. n.s. p = 0.145.

Extended Data 3

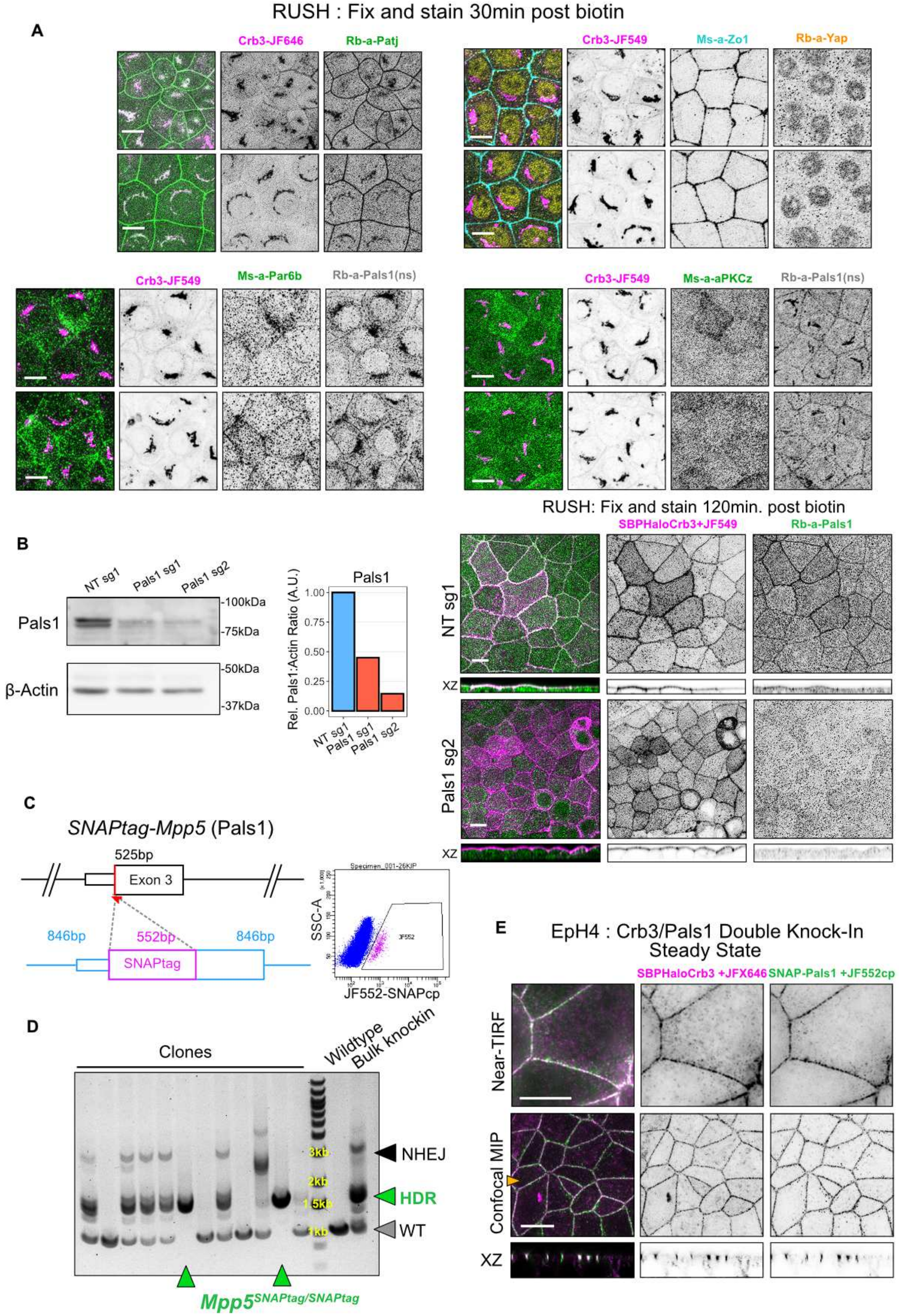

Extended Data Fig. S3.

(A) Images of SBP-HaloTag-Crb3 RUSH cells fixed after 30 minutes biotin, and immunostained against endogenous protein targets as indicated. Confocal maximum intensity projections.
(B) Left: Immunoblot of SBP-HaloTag-Crb3 RUSH cells transduced with pLentiCRISPRv2 virus with nontargeting (NT), or Pals1 sgRNAs. Right: Images of nontargeting, or Pals1 deleted SBP-HaloTag-Crb3 RUSH cells fixed after 120 minutes biotin, and immunostained against endogenous Pals1.
(C) Left: Schematic of CRISPR/Cas9 gene editing strategy to insert the SNAPtag sequence into *Mpp5* (Pals1) exon 3, the first coding exon of the gene. In red: guide RNA targeting and approximate cut site. In magenta: SNAPtag insert. In blue: homology arms flanking the insert to facilitate homology directed repair (HDR). Right: FACS gating to isolate JF552-SNAPcp+ clones.
(D) Genotyping for *SNAPtag* HDR alleles in putative knock-in clones. PCR product lengths: wild-type allele: 1054bp; HDR allele: 1606bp; nonhomologous end-joining allele: ~3300 & ~2400bp.
(E) Confocal microscopy of EpH4 $Crb3^{SBP\text{-}Halo/SBP\text{-}Halo}$ $Mpp5^{SNAPtag/SNAPtag}$ double knock-in cells at steady state labeled with JFX646-Halo and JF552-SNAPcp.

Extended Data 4

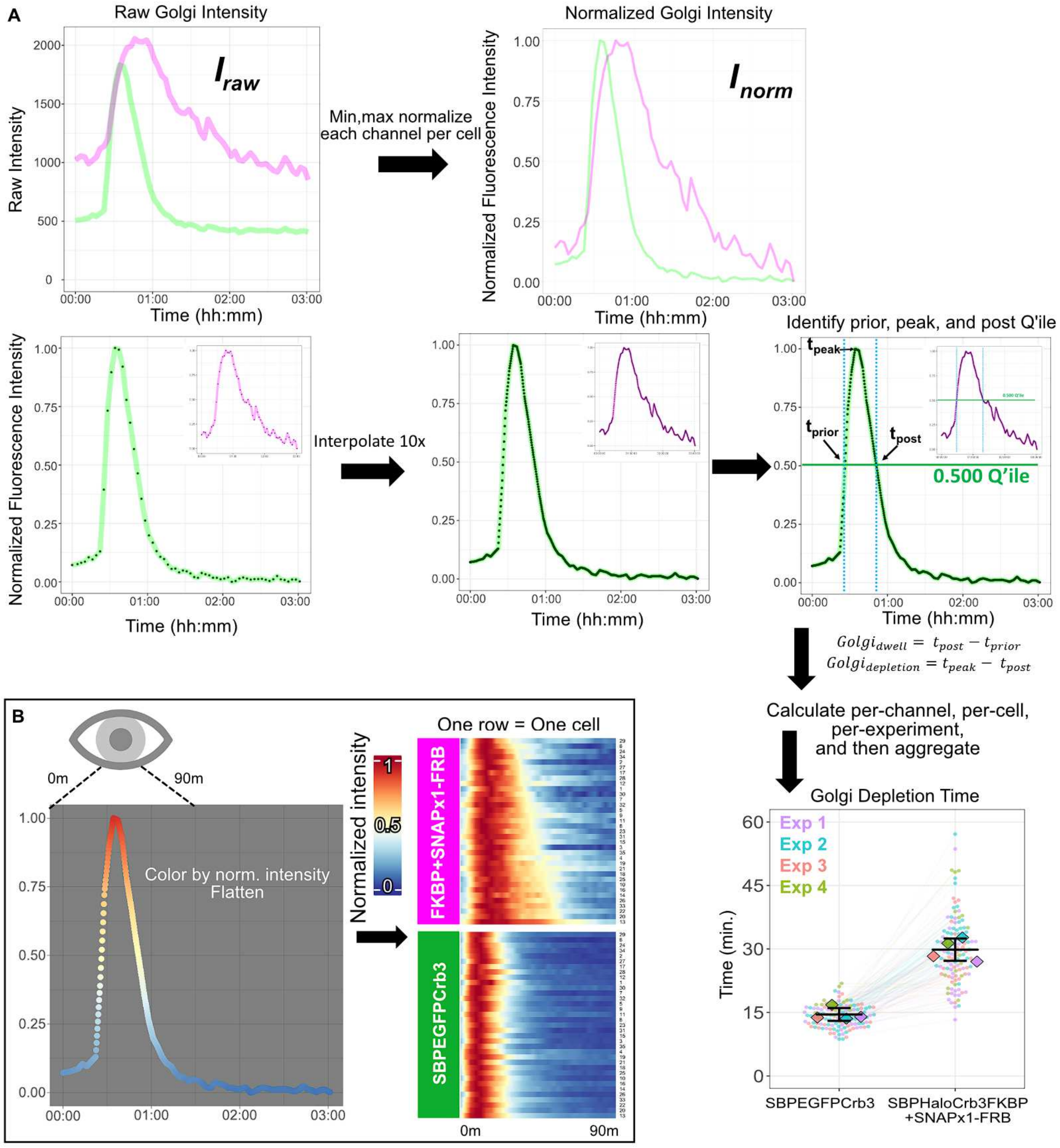

Extended Data Fig. S4.
(A) RUSH normalization and quantification strategy, as detailed in Methods: “RUSH quantification”
(B) RUSH heatmap visualization, as detailed in Methods: “RUSH Heatmap Visualization”

Movie S1.
Related to Fig. 2C: RUSH of exogenous SBP-HaloTag-Crumbs3 in polarized EpH4 cells. Top: Confocal maximum intensity projection. Bottom: Denoised XZ orthogonal view. Scalebar: 10μm. Time formatting: hh:mm:ss.

Movie S2.
Related to Fig. 2D: RUSH of endogenous SBP-HaloTag-Crumbs3 in polarized EpH4 cells. Confocal Z-slice. Scalebar: 10μm. Time formatting: hh:mm:ss.

Movie S3.
Related to Fig. 2E: RUSH of exogenous SBP-HaloTag-Crumbs3 and SBP-EGFP-Ecadherin in polarized EpH4 cells. Confocal denoised XZ orthogonal view. Scalebar 10μm. Time formatting: hh:mm:ss.

Movie S4.
Related to Fig. 3D: RUSH of exogenous SBP-EGFP-Crumbs3 and SBP-HaloTag-Crumbs3-FKBP in polarized EpH4 cells. Top: with SNAPx1-FRB and vehicle. Middle: With SNAPx1-FRB and CID. Bottom: With SNAPx3-FRB and CID. Confocal maximum intensity projection Scalebar 10μm. Time formatting: hh:mm:ss.

Movie S5.
Related to Fig. 4C: RUSH of exogenous SBP-mNeonGreen-Ace2 and SBP-HaloTag-Ace2-FKBP in polarized EpH4 cells. Top: with SNAPx1-FRB and vehicle. Middle: With SNAPx1-FRB and CID. Bottom: With SNAPx3-FRB and CID. Confocal maximum intensity projection Scalebar 10μm. Time formatting: hh:mm:ss.

Movie S6.
Related to Fig. 5C: RUSH of exogenous SBP-HaloTag-Crumbs3 in polarized EpH4 cells also expressing mApple-Pals1. Confocal maximum intensity projection Scalebar 10μm. Time formatting: hh:mm:ss.

Movie S7.
Related to Fig. 5D: RUSH of exogenous of exogenous SBP-HaloTag-Crumbs3 in polarized EpH4 cells also expressing mApple-Pals1. Note evolution of Pals1 positive, Crumbs3 negative puncta around 17:45. Denoised 3D Alpha projection. Bounding box is 60μm x 60μm. Time formatting: hh:mm:ss.

Auxiliary Material 1.
Related to Fig 1: List of polarized membrane proteins from *Zeke et al., 2021* detailing their localization, cytoplasmic and total amino acid lengths.

Auxiliary Material 2.
Spreadsheet of vector constructs, dyes, reagents, and antibodies used in this study, along with catalog numbers where applicable.
